# The Potential of Artificial Intelligence in Unveiling Healthcare's Future

**DOI:** 10.7759/cureus.71625

**Published:** 2024-10-16

**Authors:** Mousumi Khanam, Sume Akther, Iffath Mizan, Fakhrul Islam, Samsul Chowdhury, Nayla Mehereen Ahsan, Deepa Barua, Sk K Hasan

**Affiliations:** 1 Internal Medicine, Dhaka Medical College, Dhaka, BGD; 2 Internal Medicine, Institute of Applied Health Sciences, Chattogram, BGD; 3 Medicine, Shaheed Suhrawardy Medical College, Dhaka, BGD; 4 Internal Medicine, Sylhet Mohammad Ataul Gani Osmani Medical College, Sylhet, BGD; 5 Internal Medicine, Icahn School of Medicine at Mount Sinai (Queens), New York City, USA; 6 Internal Medicine, Bangladesh Medical College, Dhaka, BGD; 7 Internal Medicine, Khulna Medical College, Khulna, BGD; 8 Mechanical and Manufacturing Engineering, Miami University, Oxford, USA

**Keywords:** ai-assisted diagnostics, artificial intelligence (ai) in healthcare, ethical challenges in ai healthcare adoption, predictive analytics in medicine, rehabilitation robotics

## Abstract

This article examines the transformative potential of artificial intelligence (AI) in shaping the future of healthcare. It highlights AI's capacity to revolutionize various medical fields, including diagnostics, personalized treatment, drug discovery, telemedicine, and patient care management. Key areas explored include AI's roles in cancer screening, reproductive health, cardiology, outpatient care, laboratory diagnosis, language translation, neuroscience, robotic surgery, radiology, personal healthcare, patient engagement, AI-assisted rehabilitation with exoskeleton robots, and administrative efficiency. The article also addresses challenges to AI adoption, such as privacy concerns, ethical issues, cost barriers, and decision-making authority in patient care. By overcoming these challenges and building trust, AI is positioned to become a critical driver in advancing healthcare, improving outcomes, and meeting the future needs of patients and providers.

## Introduction and background

**Figure 1:**
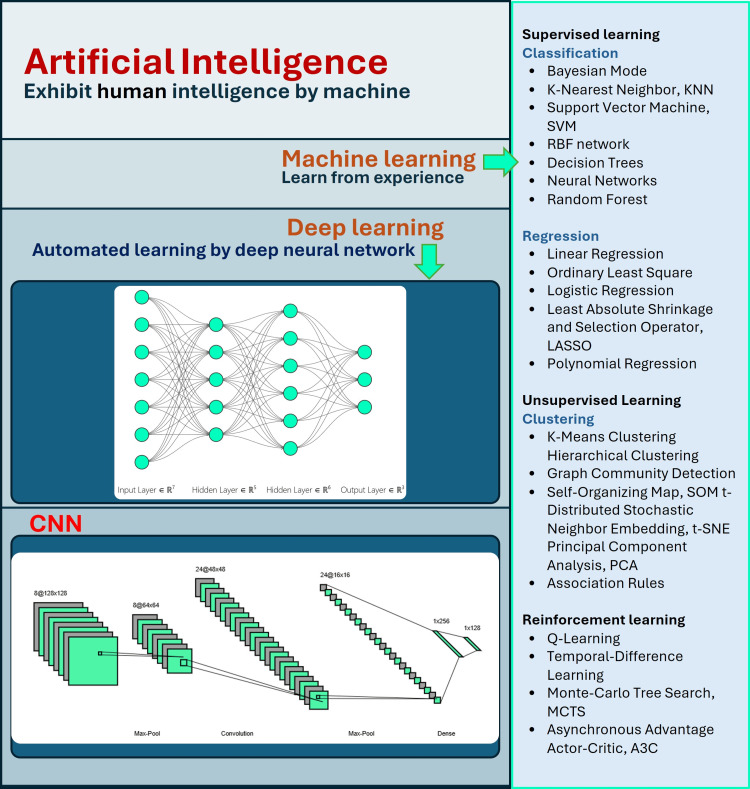
Various branches of artificial intelligence

Artificial intelligence (AI) is the field of computer science focused on creating machines and systems capable of performing tasks that typically require human intelligence. These tasks include reasoning, learning, problem-solving, understanding natural language, recognizing patterns, and making decisions. Machine learning is a subset of AI that enables systems to learn from data and improve their performance without explicit programming. It uses algorithms to recognize patterns, make decisions, and predict outcomes based on input data. The necessity for sophisticated machine learning methods to manage the enormous volumes of health data produced in contemporary medicine is a major factor contributing to AI in the healthcare industry's exponential growth. AI functions autonomously in terms of algorithms and learning procedures, but the use of several machine learning models that can handle various data formats and structures makes AI valuable. Machine learning components, including support vector machines (SVM), neural networks (NN), deep learning (DL), and convolutional neural networks (CNNs), have played a major part in the remarkable surge in research conducted in recent years on the application of AI in medical disciplines [1]. Figure 1 shows different branches of AI [2].

DL NN is one of these most frequently employed algorithms, especially in applications like medical imaging, stroke diagnosis and therapy, and early cancer and neurological illness identification. AI has proven to be more accurate in diagnosing conditions and predicting risks than conventional human techniques, highlighting its potential to transform the healthcare industry completely. However, even with these encouraging developments, there are still a lot of data- and regulation-related obstacles to overcome before AI is widely used in healthcare [3].

Several review articles have already been published on the use of AI in medical science. Among these, Basu et al. focus on the general structure of AI systems, covering key aspects such as data collection, preprocessing, model selection based on the type of data and desired outcomes (whether classification or prediction), and the subsequent training and development of the AI model for practical applications [4].

Kuwaiti et al. provide a detailed overview of AI's role in healthcare but fall short in addressing its limitations. It presents an overly optimistic perspective without fully exploring real-world challenges like algorithmic bias, misdiagnoses, and ethical issues such as data privacy. The discussion on governance and potential solutions is shallow, lacking concrete strategies to tackle these barriers. It also downplays the impact AI may have on healthcare professionals, such as job displacement and the need for training. Furthermore, the article lacks real-world case studies or empirical evidence, making its claims less practical and limiting a balanced view of AI's potential [1].

Alowais et al. explore the potential of AIs in healthcare but lack sufficient critical analysis of their limitations. While it emphasizes AI's benefits in diagnostics and treatment, it underplays significant challenges like algorithmic bias, data privacy, and the risk of over-reliance on automated systems. The discussion on ethical concerns is superficial, without offering concrete solutions for ensuring safe AI implementation. Additionally, the article could benefit from more empirical evidence and case studies to support its claims. Overall, it presents an optimistic view but falls short of addressing the complexities of integrating AI into real-world healthcare settings [5].

The article titled “Artificial Intelligence in Medical Science: A Review” provides a broad overview of AI in healthcare, but it lacks sufficient depth in addressing key challenges [6]. While highlighting AI's potential, it downplays critical issues like algorithmic bias, data privacy concerns, and the cost of AI adoption. The ethical concerns are mentioned but not explored thoroughly, leaving a gap in proposing actionable solutions. Additionally, the article underrepresents the complexities of integrating AI into real-world clinical workflows and the potential for job displacement among healthcare professionals. Overall, it presents an overly optimistic view without adequately balancing the risks and limitations.

Rajpurkar et al. provide a comprehensive overview of AI in healthcare but lack sufficient critical analysis of the practical challenges involved. It highlights AI’s potential but underplays key issues such as data privacy, algorithmic bias, and the complexity of real-world implementation. Ethical considerations, while mentioned, are not explored in depth, leaving gaps in addressing regulatory challenges and accountability. Furthermore, the article offers an overly optimistic view, with limited discussion on AI’s potential limitations, such as over-reliance by clinicians or job displacement. More empirical evidence and case studies would strengthen the article’s claims and provide a balanced perspective [7].

Ali et al. provide a thorough exploration of AI’s benefits in healthcare but lack sufficient critical engagement with its challenges. While it highlights the advantages of AI in diagnostics, patient care, and cost reduction, it downplays significant issues such as data privacy, ethical concerns, and the risks of over-reliance on AI systems. The discussion of these risks is superficial and does not offer concrete solutions or actionable frameworks for addressing them. Moreover, the article could benefit from more real-world case studies and empirical evidence to support its claims. Overall, it presents an optimistic view without fully considering the complexities of AI integration [8].

Liu et al. provide a detailed overview of AI applications in medicine but lack depth in addressing the practical challenges of implementation. While it highlights advancements in machine learning, robotics, and image recognition, it downplays key concerns such as data privacy, algorithmic biases, and the complexity of integrating AI into clinical practice. Ethical considerations are briefly mentioned, but there are no robust discussions on solutions or governance frameworks. Furthermore, the article presents an overly optimistic view of AI without sufficient real-world case studies to substantiate its claims, limiting its relevance to practical healthcare challenges [8].

Bitkina et al. present a comprehensive overview of AI's role in healthcare but has several limitations. While it effectively highlights AI's potential, it underplays the significant barriers to adoption, such as cost, ethical concerns, and regulatory challenges. The discussion on user trust and transparency, though acknowledged, lacks depth and actionable solutions. Additionally, the article provides limited empirical data and case studies, which weakens its practical applicability. Despite claiming novelty, it largely reiterates well-known trends without providing fresh insights or innovative perspectives on AI's integration into the healthcare system [9].

Through a comprehensive review of existing literature, we identified key limitations in the application of AI within healthcare. This research article aims to address these challenges by examining AI's role across various domains, including cancer screening and prevention, reproductive health, cardiology, outpatient care, laboratory diagnostics, language translation, neuroscience, robotic surgery, radiology, drug discovery, personal healthcare, and telemedicine. Additionally, we explore the barriers that hinder AI's widespread adoption in medical science, offering strategies to overcome these obstacles and improve AI's integration and effectiveness. A systematic review was conducted, and the article is organized as follows: the selection methodology, including the search strategy and inclusion criteria for the systematic review; AI applications across multiple domains; challenges and barriers; and the conclusion of the study.

Article selection methodology

A systematic review is a rigorous, structured process of synthesizing existing research evidence to address a specific research question. Unlike traditional narrative reviews, systematic reviews employ a comprehensive, transparent methodology that follows predefined protocols to minimize bias and ensure the inclusion of all relevant studies. This makes the findings of systematic reviews more reliable for informing decision-making in healthcare, policy development, and scientific research. 

*Search Strategy* 

We conducted a comprehensive literature search using PubMed and Google Scholar, using “Artificial Intelligence (AI), Medical Imaging, Clinical Decision Support Systems (CDSS), Drug Discovery and Development, Predictive Analytics and Risk Stratification, Virtual Health Assistants, Remote Monitoring and Telemedicine, Natural Language Processing (NLP), Genomics and Precision Medicine, Cancer Detection, Robotics, Healthcare Operations Management, Ethical Considerations." Initially, 94 articles were retrieved from PubMed and 27 from Google Scholar (Figure 1). The screening process adhered to the Preferred Reporting Items for Systematic Reviews and Meta-Analyses (PRISMA) 2020 guidelines to ensure no duplication.

Inclusion Criteria

**Figure 2:**
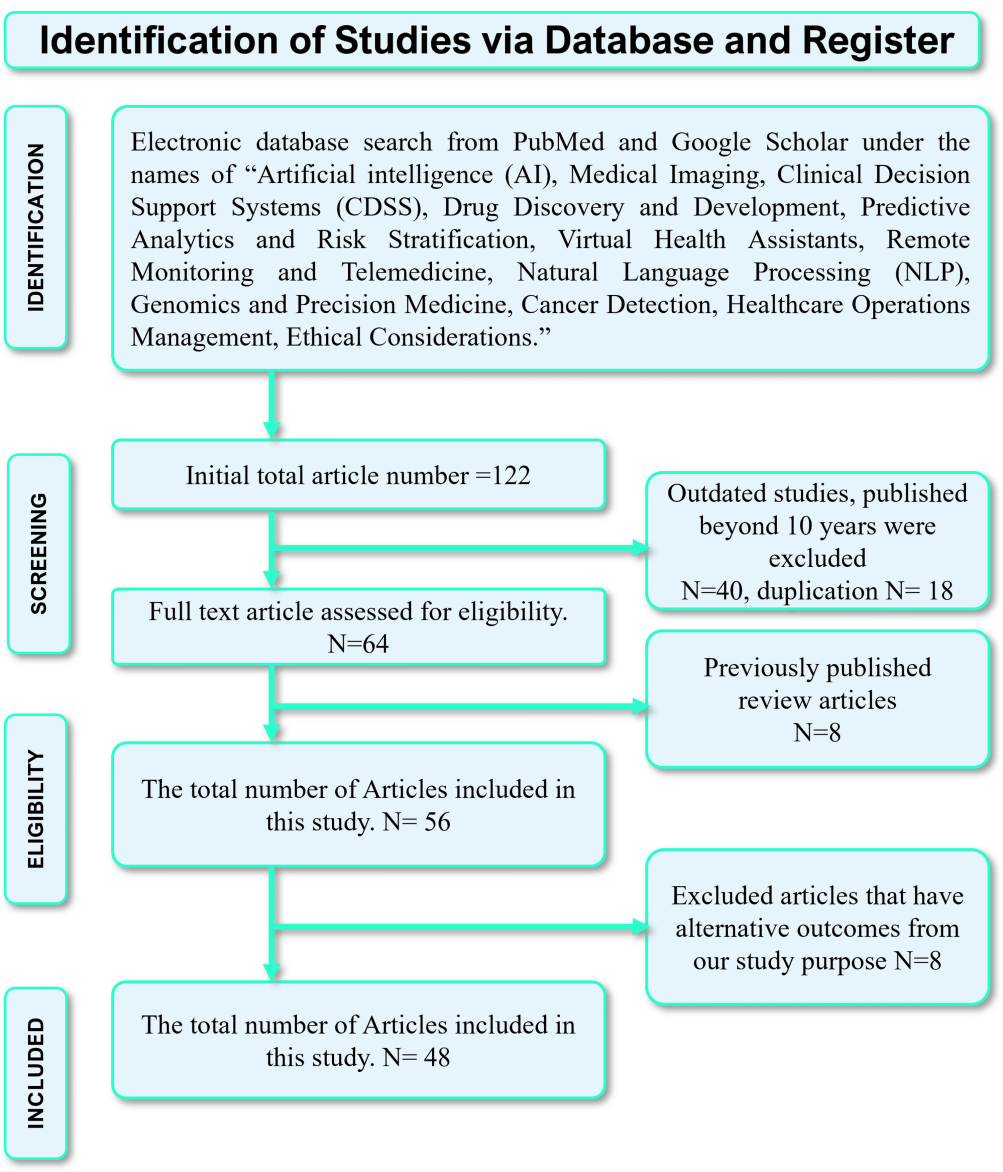
Systematic review article selection methodology

We included any prospective, retrospective, clinical, and preclinical trials meeting our inclusion criteria in the review. Subsequently, the selected studies were assessed based on inclusion criteria:  publication within the last 10 years (2014-2024), written in English, and available as full-text articles. Articles published before 2014 and articles in other languages were excluded. This process resulted in a total of 94 articles. Articles with flawed methodologies or unrelated outcomes were further eliminated. Ultimately, 37 articles were deemed relevant, with 57 review articles providing the most pertinent information, consequently included in our study. Studies not meeting the specified inclusion criteria were excluded from the study. Study findings were presented using a narrative synthesis approach. A detailed flow diagram depicting the selection of studies is presented in Figure 2. 

## Review

Applications of AI in multiple domains in medical science

The integration of AI into healthcare has led to a new era of innovation and efficiency across various medical domains. In cancer screening, AI algorithms enhance early detection and diagnosis, significantly improving patient outcomes. In reproductive health, AI facilitates personalized treatment plans and optimizes fertility assessments. Cardiology benefits from AI's ability to analyze complex cardiac data, enabling timely interventions. Outpatient care and laboratory diagnostics are increasingly streamlined through AI-driven automation and decision support systems. Additionally, AI-powered language translation tools help overcome communication barriers in diverse patient populations. In neuroscience, robotic surgery, and radiology, AI enhances precision and reduces human error. Personal healthcare is transformed through AI-driven applications that promote patient engagement and adherence to treatment. Furthermore, AI-assisted rehabilitation with exoskeleton robots represents a breakthrough in physical therapy, while administrative tasks become more efficient through automation, allowing healthcare professionals to focus on delivering high-quality care. The following section explains the individual fields in more detail.

Use of AI in Cancer

**Figure 3:**
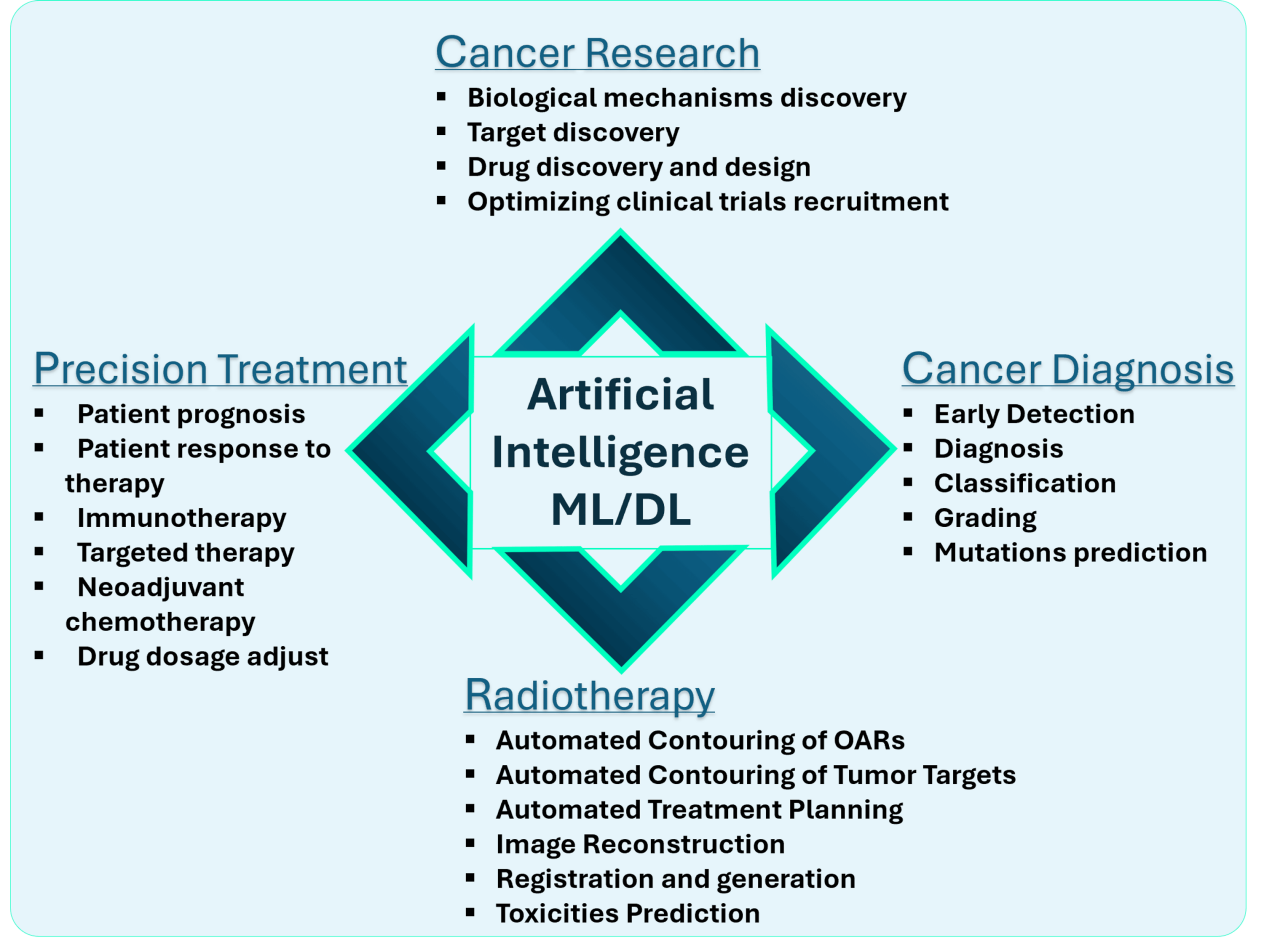
Application of AI in different branches of cancer treatment and research

AI has dramatically transformed the field of medicine over the past decade, particularly in oncology. The DL branch of AI excels in automated feature extraction, allowing it to analyze and interpret vast volumes of complex medical data. By leveraging large-scale datasets and advanced computational methods, AI has made significant strides in oncology, with applications such as molecular profiling, early cancer diagnosis, tumor classification and grading, and predicting patient outcomes and treatment responses. Furthermore, AI has improved radiotherapy procedures, facilitated personalized treatment plans, and contributed to the development of novel anti-cancer drugs and clinical trials. As AI continues to be integrated into clinical settings, it holds immense potential for revolutionizing cancer detection and treatment, heralding a new era of AI-powered cancer care [2,10]. Figure 3 illustrates the various ways AI is advancing cancer research.

Screening initiatives have led to considerable reductions in mortality rates for several prevalent malignancies. Timely interventions can lower the incidence of invasive cancers by enabling the early diagnosis of precancerous lesions, such as adenomatous polyps in colorectal cancer and cervical intraepithelial neoplasia (CIN) in cervical cancer [11]. AI is enhancing screening processes; for instance, AI-assisted dual-stained cytology for cervical cancer demonstrates greater specificity than conventional Pap smears, reducing unnecessary procedures like colposcopies. Additionally, AI has improved adenoma detection rates in colorectal cancer screening and has shown high accuracy in diagnosing breast and lung cancers [12].

AI in Reproductive Health

Comprehensive prenatal care is crucial for informed decisions and healthy deliveries, but analyzing diverse data sources poses challenges [13-15]. AI technologies are increasingly valuable for such analysis, aiding medical diagnostics and treatment decisions for pregnant women [13]. Globally, infertility affects both genders, with rising rates due to heightened awareness. AI applications in reproductive medicine have evolved significantly since the late twentieth century. It has the potential to improve infertility diagnosis and assisted reproductive technology (ART) outcomes, especially in cases of recurrent ART failure, leading to higher pregnancy and live birth rates [16]. Supervised learning methods like decision trees, SVMs, and naive Bayes classifiers are widely used for tasks such as optimizing embryo parameters, assessing cost-effectiveness in oocyte cryopreservation, and predicting outcomes in assisted reproduction [17]. Unsupervised learning models are not extensively utilized yet but show potential in predicting pregnancy based on oocyte quality with a 60% success rate [16]. Artificial NNs play a crucial role in tasks like embryo segmentation and predicting IVF success [18]. In robotic surgery within reproductive medicine, reinforcement AI is prevalent, enhancing procedures like myomectomy and male infertility operations. Robotic surgeries offer benefits such as shorter hospital stays and faster recovery, with reproductive outcomes comparable to traditional methods [19-21]. Moreover, AI is transforming male infertility assessment with automated semen analysis and could predict semen quality based on lifestyle and environmental factors. Combining AI with ART personalizes treatment, improves pregnancy rates, and reduces costs by focusing on sperm selection, embryo quality assessment, and treatment outcome prediction [16].

Use of AI in Cardiology

AI is rapidly gaining importance as a tool in cardiology, particularly in nuclear cardiology, where its applications are expanding swiftly. A key focus is on evaluating the training and testing protocols for new AI methods to ensure their clinical feasibility. AI has shown great potential to enhance image reconstruction, leading to reduced radiation exposure or improved image quality [22]. Multiple aspects of nuclear cardiology, including image reconstruction and clinical reporting, could benefit from AI algorithms. By integrating clinical, stress, and imaging data with machine learning, AI can improve the detection of obstructive coronary artery disease (CAD) and enhance risk prediction. Additionally, AI algorithms have been developed to simulate attenuation correction imaging, minimize misregistration, and improve overall image quality, making data science a critical driver of change in cardiovascular imaging [23].

The main AI applications in cardiovascular imaging revolve around image interpretation, diagnostic support, and disease phenotyping. Cluster analysis, which groups relevant imaging and clinical data, presents new opportunities for better disease characterization. Automated measurement and image segmentation tools promise to streamline diagnosis, while efforts to automate image acquisition and analysis are already underway. These advancements suggest that AI’s greatest contribution will likely be through the development of advanced precision medicine tools for cardiologists and imaging specialists [24]. Beyond imaging, AI has also introduced innovative approaches to managing hypertension. Recent studies have demonstrated AI’s ability to identify hypertensive conditions, predict incidence, and forecast outcomes. AI can estimate clinical events, particularly cardiovascular outcomes in hypertensive patients. Significant variables, such as age, systolic blood pressure (SBP), and urine albumin/creatinine ratio, have been used to predict cardiovascular diseases, with accuracy expected to increase as more health data are collected over time [25].

AI is further revolutionizing cardiac care through automated cardiovascular magnetic resonance (CMR) analysis, enhancing efficiency, reproducibility, and accuracy in assessing critical imaging biomarkers, particularly in myocardial infarction (MI). For example, AI is utilized in perfusion mapping, allowing for the rapid quantification of myocardial blood flow using CMR imaging, which offers valuable prognostic information for individuals with suspected CAD. Supervised machine learning has been employed to predict the occurrence of ventricular arrhythmia in MI patients by analyzing image-derived features such as scar texture, size, and location. Additionally, AI-driven radiomics and texture analysis have been applied to differentiate between acute and chronic MI and segment myocardial scars, further advancing the field of cardiovascular care.

Use of AI in Outpatient Settings

AI is rapidly expanding its applications in primary care (PC), with diagnostic support being one of its most critical roles. It enhances the recognition of medical images, including fundus images, X-rays, and dermatological images. Additionally, AI aids in interpreting electrocardiograms and transcribing clinical information through speech recognition tools [26].

In therapeutic support, AI personalizes medical treatments and predicts patient adherence, leading to more efficient and effective care. It also helps anticipate adverse drug reactions, thereby improving treatment safety. Moreover, AI is valuable for risk prediction, functioning as a medical triage tool that helps prioritize patients based on their needs. Personalized screening and follow-up for specific diseases based on individual risk estimates are also promising AI applications [26].

In managing chronic diseases prevalent in PC, AI has been particularly effective in cardiovascular disease management, such as arterial hypertension. Research includes early diagnosis of heart failure and identification of atrial fibrillation from ECGs. In dermatology, AI algorithms initially focused on skin cancer but now cover various skin pathologies, achieving diagnostic accuracy comparable to dermatologists. However, these algorithms need further training for conditions prevalent in PC to improve their performance. Notably, they often provide multiple diagnoses, aiding differential diagnosis in 92% of cases assessed by PC professionals [26].

In respiratory pathology, AI supports reading chest radiographs and enhances the diagnosis of pulmonary diseases like COPD and asthma by combining pulmonary function tests with clinical data. 

AI's role extends to simplifying PC consultations through speech recognition and text generation, aiding in the synchronous electronic recording of medical records and report writing. Generative AI, such as chatbots, offers patients a tool for consulting on common symptoms and receiving personalized health advice, promoting health through tailored recommendations [26].

Waiting times in outpatient clinics are a significant global issue, encompassing both actual and perceived delays. To address this, Shanghai Children’s Medical Center (SCMC) and YITU Technology Company implemented an AI system named XIAO YI, which uses DL for personalized inquisition and automatic diagnosis. This system allows patients to undergo recommended tests before seeing a doctor, thereby reducing waiting times. This study confirmed that the AI-assisted approach not only decreased waiting times but also reduced overall costs for patients, suggesting an effective method to streamline outpatient services [27].

AI and machine learning could revolutionize healthcare as significantly as electronic health records (EHR) did [28]. To ensure PC benefits from AI/ML, providers must have access to information on the populations these tools were developed and tested on, along with data definition and interoperability requirements. Standardizing this information into device labels is crucial. Given that PC accounts for most healthcare interactions, it must not be left behind in the AI/ML revolution [28].

AI in Clinical and Laboratory Diagnosis

AI has significantly transformed healthcare, improving diagnosis and treatment decisions through the use of machine learning for structured data and advanced DL and NLP for unstructured data. Key medical fields, such as oncology, neurology, and cardiology, have seen major benefits from AI advancements [29]. For instance, DL aids in the detection of potentially malignant tumors in radiographic images, while radiomics identifies critical patterns in medical imaging that may be overlooked by the human eye [30]. NLP has revolutionized healthcare processes by organizing patient records and transcribing clinical conversations, and robotic process automation (RPA) enhances efficiency by automating repetitive administrative tasks [31].

AI is reshaping the healthcare system by improving diagnostic tools and creating personalized treatment plans. These innovations allow for more accurate and individualized patient care. Furthermore, AI plays a crucial role in education and research, facilitating enhanced learning opportunities and analyzing vast datasets to uncover new insights and discoveries [5]. Looking ahead, AI has the potential to further accelerate diagnostic processes by rapidly analyzing symptoms, data, and images, ultimately improving both diagnostic efficiency and accuracy in medical practice [32].

Use of AI in Language Barrier

Patients in the healthcare system face considerable challenges due to language limitations, which can lead to inequities in service quality, safety, and health outcomes. When it comes to medical errors and outcomes, patients who encounter language problems tend to be less fortunate than those who understand English. For patients with complicated care needs, such as those nearing the end of their lives or experiencing a critical disease, these differences are particularly noticeable. Research has demonstrated that patients with language challenges and immigrants use healthcare services more frequently and have worse results in critical care settings. They frequently have lengthier stays in the intensive care unit (ICU), greater rates of death in the unit, more forceful interventions, and inadequate symptom control. Furthermore, they are less inclined to put comfort measures into place or to promptly, if at all, revive directives [33].

According to multiple studies, patients who do not have access to interpreters do poorly, which makes the lack of interpreters worse. Communication, clinical results, and patient happiness have all been demonstrated to improve when interpreters are involved. AI integration into clinical operations offers a potential remedy to these problems. AI can assist in identifying patients who require complex medical care and have language challenges, which will enhance the employment of interpreters [33].

Predictive analytics and machine learning are two examples of AI technologies that are already being utilized in healthcare for medication mistakes prevention, diagnosis, and prognosis. In healthcare, AI can help address language barriers by providing support to healthcare professionals on the use of interpreters, optimizing interpreter resources, and promoting best practices to improve health equity and enhance care quality and safety [33]. Real-time translation is made possible by innovative AI systems like NVIDIA's Riva (NVIDIA, Santa Clara, CA) and advanced language models like Microsoft's PaLM 2 (Microsoft Corp., Redmond, WA), Meta's LLaMA 2 (Meta, Menlo Park, CA), and OpenAI's ChatGPT 4 (OpenAI, San Francisco, CA). Improved medical communication and expanded access to medical education are critical roles AI plays in bridging linguistic and cultural divides in healthcare [34].

Use of AI in Neuroscience

Neuroscience is the scientific study of the brain's structure and mental processes, and it has increasingly become interconnected with AI. AI, especially through deep NNs, has revolutionized neuroscience by enabling a range of applications. Inspired by the biological NN, DL models have surpassed traditional machine learning, creating new opportunities for the study of the brain and its functions. AI has brought significant advancements to neuroimaging, enhancing both upstream tasks, such as better image acquisition, noise reduction, and image reconstruction, and downstream tasks, like detecting abnormalities, diagnosing conditions, and planning treatments.

AI has played a crucial role in analyzing complex neuroscience data, offering new ways to diagnose and predict various neurological disorders. As the collaboration between AI experts and neuroscientists continues, AI models are expected to become highly effective, reaching the point of clinical use in diagnosis and treatment. AI also supports studies at the genetic level, helping researchers analyze neurons and simulate their behavior. This allows scientists to understand how impulses in the brain are generated and transmitted through the body. Such studies are essential in identifying specific cellular traits that cause neurological diseases. For example, AI helps in studying the connectome, a detailed map of neuronal connections in the brain, providing a better understanding of brain function.

AI has also made impressive contributions to neuroimaging analysis. Deep NNs can process and segment complex brain data with accuracy similar to that of experienced radiologists. These AI systems can automatically detect and extract features from brain images, assisting in both upstream tasks, such as reducing noise and improving image resolution, and downstream tasks like diagnosing and characterizing disorders. Moreover, AI improves the efficiency of brain imaging by reducing radiation doses and enhancing image reconstruction speed. In the field of brain aging, AI has been trained to create detailed anatomical brain maps that help researchers study aging processes in the brain.

In neuro-oncology, AI aids in providing more accurate diagnostic and therapeutic options for diseases like brain tumors [35]. In stroke research, AI has led to promising advances in predicting patient outcomes and understanding the neurobiological mechanisms that influence recovery. AI’s prediction methods are proving to be superior to traditional stroke rehabilitation technologies [36]. Additionally, AI has shown the ability to detect Parkinson’s disease by analyzing nighttime breathing patterns, offering insights that no human doctor could achieve. AI can also track the severity and progression of Parkinson’s, providing new clinical insights into the disease's respiratory symptoms, such as muscle weakness and sleep-breathing disorders [37].

Use of AI in Robotic Surgery

The COVID-19 pandemic has highlighted the need to reduce contact-based treatment and increase the use of robotics in healthcare settings, particularly critical care [38]. Social robots (SRs) have gained importance in addressing challenges posed by an aging population and the pandemic, with AI accelerating their development. These robots assist in healthcare management by supporting children's education, providing personal care for the elderly (such as nurse robots), and improving mental health for individuals with disabilities (such as animal companion robots). SRs are artificial systems designed to interact positively with humans and non-humans in society.

Robotics is becoming increasingly relevant in critical care. In patient evaluation, particularly through ultrasound examinations, robots have enhanced efficiency, offering excellent outcomes. They also play a significant role in reducing costs and improving productivity in drug delivery and dispensing. In ICUs, telepresence robots have proven crucial in reducing response times, enabling quicker interventions, and lowering mortality rates [39]. Robotics, combined with telemedicine, can revolutionize healthcare by enabling remote patient monitoring, improving clinical care, and performing physical examinations from afar. The COVID-19 pandemic and Ebola outbreak demonstrated various robotic applications, including disinfection, medication delivery, vital sign monitoring, and patient communication.

Telepresence robots, which enable two-way communication between patients, families, and healthcare providers, help reduce isolation. To further integrate robotics and telemedicine, collaboration between experts in infectious diseases, engineering, and video technology is vital. However, challenges like network issues, precision, and communication difficulties must be addressed. AI and robotics are essential in minimizing exposure during infectious disease outbreaks and enabling efficient telemedicine services [40].

Use of AI in Diagnostic Precision (Radiology and Ultrasound, Emergency Medicine)

AI has shown promising results in risk assessment, disease diagnosis, and management across various medical fields, improving accuracy and efficiency. AI-powered systems can assist in clinical decision-making, helping doctors determine the best diagnosis and treatment plans while also streamlining workflows and automating processes. Compared to traditional diagnostic methods, AI offers enhanced precision at reduced costs. For instance, the Mayo Clinic's AI system for cervical cancer screening reported an accuracy rate of 91%, surpassing the 69% accuracy rate of human experts [32]. AI can process vast amounts of data from different sources, minimize human error, and continuously operate, learning to identify and classify diseases that were previously difficult to diagnose [5]. Additionally, AI-based systems can suggest potential treatment options, accelerating patient care delivery.

AI’s DL algorithms, CNNs, and data mining methods are particularly useful in diagnosing conditions such as melanoma, diabetic retinopathy, and cardiovascular diseases [41]. One notable application of AI is in image analysis, where it improves diagnostic accuracy by analyzing X-rays, CT scans, MRIs, and ultrasounds. For example, AI’s DL algorithms have demonstrated higher sensitivity and specificity in identifying pneumonia from chest radiographs compared to human radiologists [41]. AI-driven image segmentation techniques allow precise anatomical delineation, volumetric lesion measurements, surgical planning, and treatment monitoring. Companies like Google and Enlitic are developing AI-powered imaging solutions that can detect lung nodules, measure breast densities, and calculate heart ejection fractions from medical images.

AI also helps automate routine but time-consuming tasks in radiology, such as image interpretation, reporting, scheduling, and billing, allowing healthcare professionals to focus on patient care. AI systems trained on large datasets of medical scans help reduce the time and cost associated with analyzing these images. Moreover, AI-powered ultrasound is becoming a common clinical tool, capable of classifying breast tissues into benign, malignant, or normal categories with high accuracy [42]. DL techniques further enhance imaging capabilities by identifying complex patterns and improving real-time image quality during ultrasound procedures [5].

In emergency departments (EDs), AI assists in triaging patients, prioritizing high-risk cases, and reducing patient wait times. AI helps alleviate overcrowding by optimizing patient flow and making decisions about the urgency of care. It also analyzes patient records to support automated decision-making, reducing diagnostic errors and improving overall healthcare efficiency [41]. AI's ability to predict patient demand and recommend optimal treatment durations ensures more effective use of resources, potentially lowering mortality rates and shortening hospital stays.

While AI in medical diagnosis is rapidly advancing, it still faces challenges such as overdiagnosis of minor conditions, high production and maintenance costs, and the need for supervision by healthcare professionals [43]. These hurdles must be addressed before AI systems can achieve widespread clinical adoption.

Use of AI in Drug Discovery and Development (Pharmacy)

The ongoing advancements in AI technology, coupled with the expanding pool of data resources, are significantly accelerating and enhancing various stages of drug discovery, like target identification, virtual screening, and drug optimization [44].

AI-driven methods, like machine learning and network-based analyses, have accelerated the identification of new targets associated with different diseases. By analyzing vast datasets, integrating diverse data sources, and utilizing advanced algorithms, AI can predict potential drug targets, recognize hit and lead compounds with remarkable accuracy, and thus assist in drug development [45].

AI is mainly used to find specific and potent candidate molecules in drug discovery and development [43]. Virtual screening refers to computationally sifting through extensive chemical libraries to select molecules with desired pharmacological character [44]. AI-powered virtual screening techniques, such as molecular docking and DL approaches, have notably broadened the chemical space for exploration, uncovering molecules that traditional screening methods might have overlooked [44]. This enables faster and cheaper identification of potential drug candidates.

AI is used in optimizing lead compounds to enhance their drug-like characteristics and safety. Reinforcement learning and generative adversarial networks (GANs) are two examples of generative models that can generate novel chemical compounds with desirable therapeutic characteristics and increase the likelihood of success in clinical trials [44]. AI-driven optimization algorithms are able to predict probable side effects and off-target interactions, which helps to develop safer and more effective drugs.

Although the use of AI in drug development is still in its early stages, there are numerous examples that demonstrate the technology's promising future. Pfizer is using machine learning-based IBM Watson to assist in the discovery of immuno-oncology medicines [46]. AI system from GNS Healthcare in Cambridge, Massachusetts is being used by Roche affiliate Genentech to help in its hunt for cancer treatments [46]. DeepMind developed the AlphaFold platform, which uses DNNs to predict 3D protein structures more accurately than other algorithms [47].

Overall, AI makes the complicated and challenging task of drug discovery and development faster, cheaper, and more effective while minimizing repeated work by simplifying the procedure. Besides aiding in various stages of drug development, AI technologies also play a promising role in repurposing (repositioning) current drugs for new potential uses [48]. The capability of AI to predict the interaction between drug and target is utilized for this purpose. AI has also shown potential in vaccine development, yielding new antibiotics, defining the mechanism of drug toxicity, and many other areas of the vast field of pharmacy.

Use of AI in Developing Personalized Healthcare

AI is gaining strong support for its potential in primary healthcare. Its ability to enhance human capacities in patient care management, diagnostics, and treatment is driving its increasing use across the healthcare industry. AI systems offer several advantages, such as improving clinical decision-making, speeding up diagnoses, and increasing accuracy while expanding predictive capabilities. They also streamline processes like chart review and documentation, optimizing operational efficiency and resource allocation. By improving care quality and continuity, AI can also enhance the patient-physician interaction, leading to better outcomes overall [49].

However, while AI excels at processing data and predicting outcomes, it cannot fully replace the complex techniques used by clinical specialists. Human expertise remains critical in interpretation and clinical judgment, as AI lacks the contextual awareness and intuitive knowledge necessary for making complex medical decisions. Moreover, AI cannot replicate the emotional and empathetic aspects of patient care, which are essential for building trust and providing comfort. Thus, AI enhances accuracy and efficiency in healthcare but cannot replace the expert knowledge and compassionate care that healthcare professionals provide [26].

AI-Driven Robot-Assisted Rehabilitation

AI and robot-assisted rehabilitation are revolutionizing physical therapy by enhancing the precision, personalization, and effectiveness of recovery processes. AI-driven rehabilitation systems often incorporate devices like exoskeletons and end-effector robots to support patients with mobility impairments, neurological disorders, or those recovering from surgeries or injuries. Rehabilitation exoskeletons are wearable robotic devices that assist or resist movement, helping patients regain strength and motor control by providing adaptive, controlled support based on real-time feedback [50,51]. AI algorithms analyze patient data, such as gait patterns and muscle strength, and adjust the exoskeleton’s movements to optimize the rehabilitation process, ensuring personalized therapy tailored to each patient’s needs [52].

End-effector robots, which focus on specific limbs or joints, are also controlled by AI systems to facilitate repetitive and targeted movement training. These robots interact with the patient’s extremities, such as hands or feet, to guide movements, promoting neural plasticity and motor learning. AI-powered control systems enable these robots to adjust resistance, speed, and range of motion dynamically based on the patient's progress or limitations. Other devices, such as robotic treadmills and AI-driven virtual reality systems, further enhance rehabilitation by offering immersive, interactive environments for patients, fostering engagement and motivation. Overall, AI’s role in controlling these devices ensures continuous, data-driven improvements in therapy, making rehabilitation more efficient and effective.

Use of AI in Overcoming Distances

The COVID-19 pandemic accelerated the adoption of telemedicine, which leverages medical information to enhance patient health remotely [40]. Telemedicine offers numerous benefits, particularly in enabling the efficient and equitable distribution of healthcare resources. It addresses the needs of regions lacking medical professionals and reduces travel-related carbon emissions. Additionally, telemedicine expands access to specialized care, which is crucial for patients with rare conditions or mobility issues. By increasing the capacity to treat both acute and chronic diseases, it also reduces waiting times and provides timely professional care, even in underserved areas [53].

Doctors can maximize their time treating patients by quickly assessing them, identifying infection symptoms, and documenting high-risk cases during teleconsultations. However, the interdisciplinary nature of diagnosis, which often requires multiple tests and consultations, presents challenges, particularly regarding the availability and cost of certain tests in telemedicine settings. Data-driven AI algorithms offer promising solutions to this complexity by utilizing large datasets for machine learning, improving diagnostic accuracy and generalizability across diverse populations. Furthermore, the integration of telepathology, tele-oncology, and teleradiology fosters more comprehensive patient care [53].

Digital consultations now allow patients to seek medical advice for minor issues from home, reducing hospital visits. AI-driven apps like Babylon and Buoy use symptom-checking tools that combine general medical knowledge with patient histories. In the UK, Babylon is widely used for online consultations, particularly by younger adults who are less likely to have a regular doctor. Buoy, launched in 2015, uses AI to assess the severity of health conditions and offers interactive symptom checks via chatbots, guiding users to appropriate care and nearby providers [54].

However, AI in healthcare faces challenges, particularly when human intuition and subjective judgment are critical. Studies, such as Jarrahi’s work on cancer diagnosis in lymph node imaging, show that AI-human collaboration can reduce errors, highlighting the potential for improving diagnosis accuracy and patient outcomes in telemedicine services.

Challenges and barriers to the implementation of AI

The integration of AI in healthcare and other industries holds great promise, yet it faces several challenges that hinder widespread adoption. These challenges are multifaceted, spanning from security concerns to ethical dilemmas. Security Barriers involve risks associated with the protection of sensitive data from cyber threats and unauthorized access. Technological Barriers include the complexity of developing and deploying AI systems that are both accurate and reliable across various applications. Furthermore, Liability and Regulatory Barriers pose legal challenges, as determining accountability for AI-driven decisions remains unclear under current frameworks. Ensuring Patient Safety is also a critical concern, as errors or misinterpretations by AI systems could have serious consequences in medical and other high-stakes environments. The Ethical Issues of Using AI involve questions about bias, fairness, and the potential for machines to replace human roles, raising concerns about transparency and trust. AI's Social Impact is another significant consideration, as automation and AI adoption may disrupt job markets and exacerbate inequality. Finally, Data Privacy concerns, particularly regarding the use of personal data in AI algorithms, create obstacles due to strict privacy laws and the need for secure data handling protocols. Addressing these challenges is essential for the safe and effective implementation of AI. The following section will explain individual sections in more detail.

Practical Barriers to AI Adoption in Healthcare Settings

The practical barriers to AI adoption in healthcare settings are multifaceted, involving technological, organizational, and regulatory challenges. One of the key obstacles is the lack of high-quality, standardized data needed for AI algorithms to function effectively. Healthcare data often exist in fragmented, unstructured formats across different systems, making it difficult to integrate and analyze at scale. Additionally, there are interoperability issues between AI systems and existing hospital infrastructures, which can hinder seamless implementation. Another challenge is the significant cost and complexity of deploying AI technologies, which require both substantial financial investment and technical expertise. Healthcare professionals often lack the necessary training to interact with AI tools effectively, leading to resistance or misapplication. Furthermore, regulatory frameworks for AI in healthcare remain underdeveloped, creating uncertainty around issues like liability, accountability, and compliance with data privacy laws. These factors, combined with ethical concerns, such as algorithmic bias and the potential displacement of healthcare jobs, make the widespread adoption of AI in healthcare a complex and gradual process.

Security Barrier

When implementing AI in the medical field, security is crucial and demands careful consideration. Because AI products depend on electronic devices like computers and mobile phones, hardware security issues come up. The physical vulnerabilities of these devices are caused by electromagnetic interference, temperature changes, and cost. Challenges may arise from the professional hurdles and complexity separating IT and medical expertise. Retraining engineers can cause productivity disruptions, and physicians frequently have difficulty using AI. As assaults on crucial nodes have the potential to cause global cascading failures, network security is also essential.

Another big worry is software security since AI algorithms can be attacked by malicious designers. Even though they perform well at first, they may struggle in focused conflicts. Attackers who fully understand the workings of an NN might produce false positives or negatives, which lead to classification errors [55].

Technological Barrier

The main obstacles to the application of AI in healthcare are those pertaining to technology. Because AI is primarily dependent on the data it is trained on, there are a number of key challenges related to data quality, accuracy, and dataset size. Problems like biases, inaccurate phenotyping, and the "black box" issue show how difficult it is to apply AI in healthcare in a reliable manner. Moreover, there are difficulties in successfully implementing AI in healthcare settings due to interoperability, usability, and integration into current workflows.

The lack of reliable evidence and prospective data makes it difficult to validate AI's effectiveness, particularly in real-world situations. Widespread AI adoption is further hampered by infrastructure and hardware limitations, as well as by the absence of recognized leaders and standards in the healthcare AI space. The obstacles to integrating AI are compounded by security concerns, notably with regard to cyber-attacks and data breaches [56].

Liability and Regulatory Barrier

Healthcare professionals are subject to stringent evaluations and are expected to follow conduct guidelines. There are concerns about whether AI is overused because there are no globally harmonized laws for AI in medicine. It is imperative that comprehensive and precise AI regulations be created, involving stakeholders in the field of AI development. It is necessary to clarify who is responsible for AI-related problems, as it may be the manufacturer, user, or maintainer. Statutes need to be amended often since health-related data have outgrown the original privacy protection provisions of statutes like Health Insurance Portability and Accountability Act of 1996 (HIPAA). There is no distinct regulatory body, despite the fact that new regulations govern AI data protection and accountability [55].

Patient Safety Barrier

By lowering mortality and increasing diagnostic precision, AI is supposed to increase patient safety, but there are hazards that need to be properly addressed as well. "Distributional shift" is a significant problem because, in contrast to clinicians who can effectively manage uncertainty and consider the implications of decisions, AI finds it difficult to deal with variances in real-world data. AI systems frequently concentrate on a single ailment, possibly overlooking related illnesses and producing inaccurate forecasts without taking the specific context into account.

Another worry is automation bias, when medical professionals may rely too much on AI, taking its advice for granted and ignoring supporting data. As algorithms become older and are unable to adjust to new medical discoveries, treatment methods, and diseases, bias may increase. Due to a lack of previous data, established AI algorithms may also be resistant to new medical advancements, preventing their safe integration into medical practice. AI systems are always learning and getting better, but there are concerns in the healthcare industry. By using patterns unrelated to actual medical expertise, AI may provide short-term results that are at odds with long-term health objectives. It is critical that AI systems evaluate the confidence in their predictions and incorporate failsafe mechanisms. In order to preserve patient safety and faith in the technology, an "explainable AI" is required to enable decision interrogation and evaluate diagnosis confidence in real time [43].

Ethical Issues of Using AI

Informed consent: A major ethical and legal challenge with the use of AI in healthcare is informed permission, which is especially problematic when it comes to imaging, diagnosis, and surgery. Healthcare professionals need to handle the challenges of teaching patients about AI, which include the various forms of machine learning, data sources, and possible biases. Transparency and accountability are hampered by the employment of "blackbox" algorithms, which are challenging to comprehend. Physicians must strike a balance between patient privacy and AI's efficacy and safety, especially in cases when patients are unwilling to divulge specific information. All things considered, as AI continues to change the patient-clinician relationship and clinical decision-making processes, creating thorough standards to address these ethical issues is essential [57].

Social Impact

The clinical relationship is anticipated to be impacted by ambient intelligence in healthcare, potentially changing the conventional doctor-patient relationship. It might participate in medical interactions as a third party or affect clinical judgment by using default settings established by healthcare institutions. This might erode patient confidence and normalize ongoing healthcare surveillance, which would have an effect on the employer-employee relationship by changing expectations regarding trust and obligations. It might lessen the pressures on caretakers, but it might also have an impact on the obligations and risk exposure of professionals.

Applications of ambient intelligence must be carefully examined in order to detect any unexpected repercussions. If other organizations utilize long-term surveillance and extensive sensor data processing to track or identify movements, this could provide ethical challenges. With the growing requests for accountability, developers and users must take responsibility for the outcomes of the system [56].

Data Privacy

The UK Information Commissioner's Office (ICO) discovered in July 2017 that the Royal Free National Health Service (NHS) Foundation Trust had breached the UK Data Protection Act 1998 by giving Google DeepMind access to the personal information of about 1.6 million patients without giving them enough notice. The "Streams" app (Google DeepMind, London, UK), which helps diagnose acute kidney injury but does not employ AI, was clinically tested using the data. The Information Commissioner stressed that basic privacy rights should not be sacrificed for innovation. This instance emphasizes how crucial it is to educate patients about data processing in order to foster their faith in AI-powered medical solutions. Successful AI integration requires trust, as demonstrated by prior cases such as Dinerstein v. Google and Project Nightingale, which also posed a risk to patient privacy. An additional crucial problem is data ownership. The public finds it unpleasant that governments or businesses would sell patient data for financial gain, despite the fact that health data can have significant value. One possible strategy to help patients feel appreciated is to emphasize reciprocity over ownership. For example, in exchange for patient data, the Royal Free NHS Foundation Trust was given five years of free usage of the Streams app. Robust safeguards are also required to stop the exploitation of patient information for objectives other than the doctor-patient relationship, such as insurance or job. Patients should also be able to request the removal of personal data, even if it has already undergone aggregate analysis [56].

## Conclusions

In conclusion, the integration of AI into healthcare presents a significant opportunity to enhance patient outcomes and revolutionize medical practices across various domains. As highlighted, AI's capabilities in diagnostics, personalized treatment, and patient management offer innovative solutions to longstanding challenges within the healthcare system. By streamlining processes such as cancer screening and drug discovery, AI not only improves efficiency but also paves the way for more accurate and timely interventions. However, realizing this potential requires a concerted effort to address the challenges associated with AI adoption, including privacy concerns, ethical considerations, and the need for regulatory frameworks. Building trust among healthcare professionals and patients is paramount, as is ensuring that AI technologies are developed and implemented with transparency and accountability. By prioritizing these efforts, we can harness the transformative power of AI to create a more effective, equitable, and responsive healthcare system. The future of healthcare, enriched by AI, promises improved health outcomes and enhanced patient experiences, ultimately fulfilling the evolving needs of both patients and providers. Embracing AI is not just a technological shift; it is a step toward a more sustainable and innovative approach to health and well-being.
